# Caregiver Peer Support for Families of Autistic Children in France

**DOI:** 10.1111/cch.70123

**Published:** 2025-07-02

**Authors:** Jeffrey McCrossin, Lucyna Lach, Sophie Biette, Émilie Cappe

**Affiliations:** ^1^ McGill University Montreal Canada; ^2^ Adapeila Nantes France; ^3^ Laboratoire de Psychopathologie et Processus de Santé Université Paris Cité Boulogne‐Billancourt France; ^4^ Institut Universitaire de France (IUF) Paris France

**Keywords:** autism spectrum disorder (ASD), caregiver peer support, family well‐being, France, neurodevelopmental disabilities (NDD), support programmes

## Abstract

**Background:**

Caring for autistic children presents significant emotional and practical challenges, impacting family well‐being and creating a need for social supports. In France, these challenges are compounded by historical reliance on psychoanalytic approaches to autism care. This study aims to identify caregiver peer support programmes for families of autistic children in France and explore their contributions to autism care.

**Methods:**

An environmental scan was conducted to identify relevant caregiver peer support programmes. This approach involved an internet search using keywords related to autism, caregiver peer support and France, supplemented by informal consultations in the autism support community to contextualize findings and refine the identification of programmes. Inclusion criteria focused on services offering ongoing support to caregivers by peers. Data extraction was performed on publicly available sources and through direct correspondence with service administrators.

**Results:**

Sixteen organizations were identified, offering various forms of caregiver peer support, including group meetings and individual follow‐ups. Most services were in‐person, with some online options. Peer support roles varied between paid and volunteer positions. Few organizations reported offering training to peers. Key support formats included informal gatherings like discussion groups focusing on emotional support, sharing experiences and advocacy. However, inconsistencies in the availability of detailed information about services and peer training highlight limited clarity in programme descriptions, which may pose challenges for families seeking support.

**Conclusions:**

Caregiver peer support is underdeveloped in France. Insufficient detail regarding service delivery models, support structures and training hinders broader accessibility and adoption. Aligning terminology and promoting the value of caregiver peer support could enhance its role in autism care. Future research should evaluate the impact of these programmes on caregiver and family outcomes and explore stakeholder experiences to refine support mechanisms.

**Summary:**

- Caregiver peer support for autism is underdeveloped in France. Despite its recognized value in improving family well‐being, these services remain limited and inconsistently implemented across the country.
- Many programmes lack peer support training for caregivers, highlighting opportunities to improve the quality and sustainability of the support provided to families of autistic children.
- There is significant variation in how caregiver peer support services are offered, ranging from informal gatherings to individualized support, with a recent shift towards professionalizing the role of peer supporters.
- Standardizing terminology, promoting caregiver expertise and researching programme outcomes could enhance the impact of caregiver peer support on family well‐being and autism care in France.
- The unique challenges and developments in France's caregiver peer support system offer valuable insights into the broader processes and evolution of peer support on a global scale.

Caring for autistic children and those with related neurodevelopmental disabilities (NDD) often presents numerous emotional and practical challenges, impacting the well‐being of families and creating a need for social supports (Desquenne Godfrey et al. 2024; McStay et al. 2014). In France, these challenges have been exacerbated by a historical reliance on psychoanalytic approaches, which delayed the adoption of evidence‐based care and marginalized families by blaming them for their child's condition (Bates 2020; Bishop and Swendsen 2021; Davidson 2014). These practices excluded caregivers from meaningful roles in their child's care, fostering a culture where families were seen as part of the problem rather than essential partners.

Parent associations, which emerged in the 1950s, have played a pivotal role in supporting families through advocacy, public education and service development. Networks such as Autisme France, Sésame Autisme and Unapei have established and managed centres and services for education, vocation and housing, helping to inform policy and provide vital resources to families (Chamak 2008). These associations were instrumental in pressuring the government to modernize autism care, which resulted in legislative changes, such as recognizing autism as an NDD and expanding access to rights and resources for families (Chamak 2008; Stratégie nationale 2023–2027 pour les troubles du neuro‐développement, 2023). While these associations have supported families for decades, their activities are not explicitly labelled as caregiver peer support, reflecting a broader delay in the development of structured peer support systems in France (Gardien and Laval 2019; Le Callonnec et al. 2022).

Caregiver peer support involves the sharing of empathic understanding and experiential knowledge among caregivers facing similar challenges, within a supportive and nonjudgemental environment (Sunderland, et al. 2013). It has demonstrated a positive impact on the well‐being of caregivers and families of children with NDDs by leveraging collective expertise to navigate challenges and access meaningful services (Chakraborti et al. 2021; Lee et al. 2023; McCrossin and Lach 2023; Pollock et al. 2022; Postma et al. 2024; Shilling et al. 2013). By drawing on caregivers' lived experiences, caregiver peer support enhances the perceived credibility of advice and authenticity of the emotional validation they offer (Bray et al. 2017; McCrossin and Lach 2023; Pollock et al. 2022; Shilling et al. 2015b; Thomson et al. 2015). Through processes of family resilience, peer support facilitates access to both internal and external family resources and enhances communication between families and care teams, thereby advancing family‐centred care delivery (McCrossin et al. 2022; McCrossin and Lach 2023; Pollock et al. 2022; Ungar 2010).

The national strategies for NDD care in France have encouraged broader adoption of evidence‐based approaches, early detection, social inclusion and the involvement of families as partners in care, including through peer support (Haute Autorité de Santé 2014; Sankey et al. 2019). However, gaps remain in recognizing and addressing caregivers' needs. Insights from the Interministerial Delegation for Autism and Neurodevelopmental Disorders (DIA‐TND) recurrent survey highlight the extent of these gaps (d'Eyssautier 2023). In 2021, among over 14 000 respondents, 4349 were caregivers. Findings revealed that 76% of caregivers reported feeling ‘blamed by certain professionals’, only 55% of caregivers reported being directed to follow‐up services after their child's diagnosis, and 62% were not part of a parent association for ASD or other NDDs. Notably, 82% relied on the internet to learn about their child's diagnosis, and 53% turned to social media or other forums for additional information. These results suggest that current services, including parent associations, may not be adequately reaching or meeting the needs of many families with children with NDD.

Systemic barriers continue to limit the integration of caregiver perspectives into support structures. For example, the network of publicly funded Mutual Support Groups (Groupes d'Entraide Mutuelle or GEMs), which are designed to promote social reintegration for individuals with disabilities and mental health conditions, exclude families from participation (Montreynaud 2019). This exclusion perpetuates the historical marginalization of caregivers, highlighting the need for caregiver peer support systems that leverage lived experiences to foster resilience and improve family outcomes.

The present study aims to identify caregiver peer supports available for families of autistic children in France and explore how this type of support contributes to the landscape of autism care in the country. To the best of our knowledge, this research is pioneering in its investigation into the distinctive features of caregiver peer support in this context.

Our specific research questions are as follows: (1) What caregiver peer supports are available for families of autistic children in France, and what is their expressed purpose? (2) Who are the target audiences of these supports (e.g., diagnosis, region)? (3) What are the key structural and operational characteristics of these supports, including their format (e.g., one‐to‐one or group, paid or voluntary) and mechanisms for sustainability (e.g., training and supervision support)?

## 1 Methodology

### 1.1 Environmental Scan

To address our research questions, we conducted an environmental scan to identify caregiver peer support programmes for families of autistic children in France. Environmental scans, originating from the business sector, are increasingly used in health services and social research to synthesize data from diverse sources, providing insights into contextual factors relevant to decision‐making and programme development (Charlton et al. 2021; Graham et al. 2008). This approach is particularly suited to exploring underresearched areas and informing programme planning by drawing on publicly available data and expert insights.

The purpose of the scan was to map existing programmes and resources, focusing on their key features, accessibility and relevance to caregiver needs. The process involved both online searches and informal consultations. Publicly available online resources, such as government websites, community organization platforms and reports, were reviewed using the search approach described below. Informal conversations with local experts and stakeholders in the autism support community further guided the identification of relevant websites and organizations.

### 1.2 Internet Search

We conducted a search in the spring of 2024 using Google as a search engine to identify potential caregiver peer supports. Keywords, developed in English and translated to French, included those related to autism spectrum disorder (ASD), caregiver peer support and France (Appendix I). We also reviewed websites related to ASD, such as resource directories and general caregiver organizations (Appendix II) to identify caregiver peer support resources potentially frequented by families of autistic children.

To minimize bias and ensure a comprehensive identification of caregiver peer support programmes, the research team employed an iterative and collaborative search process. The first author conducted the initial search using predefined terms developed in consultation with the research team. As results emerged, additional terms frequently identified in the resources were incorporated to refine the search.

Identified resources were regularly reviewed by the research team and community experts to validate their relevance and identify potential gaps. Community experts, familiar with the local family support landscape, provided insights to expand the search and ensure its comprehensiveness. This iterative review and feedback process helped limit bias and maximize the scope of identified programmes.

### 1.3 Inclusion and Exclusion Criteria

Resources, in English or French, were included for data extraction if services were directly offered on a regular basis to caregivers or family members of autistic children by peers (e.g., individual peer support) and/or in a peer environment (e.g., peer support group). Resources were not excluded if they served broader populations with related NDDs rather than focusing on solely on autism. Only resources advertised online with ongoing and/or regularly scheduled activities were included. Organizations offering only one‐off events were excluded from this scan.

### 1.4 Data Extraction

We extracted publicly available data from relevant websites using the data extraction table (Appendix III). Missing data were sought through correspondence with administrators of the respective organizations. Data were thematically analysed to identify patterns, gaps and variations in programme features and implementation contexts.

## 2 Results

The search identified numerous organizations, including over 100 from an online service directory (https://www.tamis‐autisme.org/) listed under parent associations reportedly offering opportunities for exchanges between individuals with autism and/or their caregivers. Reasons for exclusion were predominantly insufficient focus on caregiver peer support (e.g., programmes centred solely on professional‐led interventions) or lack of publicly available details about their current support offerings.

A total of 16 organizations were identified and included for data extraction. Of these, six programmes were specifically designed for families of children with ASD or other NDD, while the remaining 10 served families of children with any type of disability. The organizations represented 14 of the 101 French departments (geographic administrative divisions). Two organizations, Cœur de Maman and Autisme Info Service, were associated with support services offered across France.

While caregiver peer support services were clearly identified across the 16 organizations (Table 1), a significant gap in publicly available information was evident. Few organizations provided comprehensive details on the delivery mechanisms for peer support, the specific qualifications or training of peer supporters or the conditions under which support was offered. For example, only five organizations explicitly reported training their peer supporters, and information about the content, frequency or impact of this training was sparse. Similarly, eight programmes clarified whether peers were volunteers (*n* = 4) or paid employees (*n* = 4), but further details on compensation structures or role expectations were lacking.

**TABLE 1:** Summary of a selection of organizations offering peer support programs or services in France.

Organization	Target condition or diagnosis	Location (department code)	Name of peer support service	Format	Meeting location	Voluntary or paid peers	Support for peer mentors
Affiche la couleur	Any disability	Indre (36)	Soutien aux parents	Individual or group	Online or in‐person	Voluntary	
Apei Ouest 44	Any disability	Loire‐Atlantique (44)	La mise en lien avec les pairs	Individual	In‐person		
Association Détours	ASD	Les Ulis (91)	Group de parole	Group	In‐person	Facilitated by professional	N/A
Autisme info service	ASD or NDD	Across France	S'informer et accompagner	Individual	Phone or online	Paid	Training in active listening and education on NDDs; access to specialized lawyer for guidance on user rights; peer support among employees for complex user situations.
Café Autisme	NDD	Toulouse (31)	Café des parents	Group	Online	Voluntary	
Centre ressources autisme Normadie‐Seine Eure (CRANSE)	ASD	Normandie (76), Seine‐Eure (27)	Parent‐ressources Autisme	Individual or group	Online or in‐person	Paid	Resource parents complete a training programme based on recommended best practices for autism care by the Haute Autorité de Santé (HAS).
Cœur de Maman	Any disability	Across France	Group de parole	Group	In‐person		
Constellation	Any disability	Loire‐Atlantique (44)	La pair‐aidance (les intervants‐pairs)	Individual or group	In‐person	Paid	Training in peer support
Espairs	Mental health or NDD	Lyon/Rhône (69)	Pair‐aidance	Individual or group	Online or in‐person	Paid	Training in peer support
Halte Pouce	Any disability	Montpellier (34)	Paroles partagées	Group	In‐person		
Handimômes Cambrésis	Any disability	Cambrésis (59)	Temps d'échange entre parents	Group	In‐person	Facilitated by professional	N/A
Ikigai	NDD	Paris (75)	Café des parents	Group	Online	Voluntary	None
L'Esperluette	Any disability	Toulouse (31)	Café des parents, « et moi alors » (sibling support group)	Group	In‐person	Voluntary	
Les Bobos à la ferme	Any disability	La Madelaine‐sous‐Montreuil (62)	Pôle parents aidants	Group	In‐person	Paid	Training offered for peer support mentors: Pairs Aidants Pro
Soliane	Any disability	Marseille (13)	Café des parents	Group	In‐person		
Tom Enfant Phare	Any disability	Agen (49)	Café des parents	Group	In‐person		

Abbreviation: ASD = autism spectrum disorder.

The variability in service descriptions (Table 2) reflects broader challenges in clarity around the supports being offered. Some organizations, such as CRANSE (Centre ressources autisme Normandie‐Seine Eure) and Constellation, detailed robust models including one‐on‐one and group‐based support, peer training and clear role definitions, while others, like Café Autisme, provided only a minimal description of their peer support group. The name of the peer support service varied considerably, though ‘café des parents’ and ‘groupe de parole’ were among the most common, which generally referred to informal gatherings where caregivers could meet and discuss their experiences, share advice and in some cases receive educational offerings from invited professionals. Some programmes focused on specific themes including supporting caregivers to advocate for educational and social inclusion for their children, while others emphasized discussion of the caregiver experience (as opposed to, e.g., supporting caregivers to address their child's concerns). Most services were offered in‐person, though six organizations indicated some level of support being offered online. Ten organizations offered only group‐based support, two provided individual peer support exclusively, and four advertised both individual and group supports.

**TABLE 2:** Website and description of peer support service or programme of identified organizations.

Organization	Website	Description of peer support program or service
Affiche la couleur	https://affichelacouleur.fr/soutien‐aux‐parents/	Phone and online support offered by two peer volunteers. A group meeting on demand is proposed.
Apei ouest 44	https://www.apeiouest44.fr/lassociation‐2/le‐service‐daide‐aux‐aidants/nos‐interventions/	Professional service that includes connecting families to peers.
Association détours	https://www.facebook.com/detours91/	Occasional group meeting for parents facilitated by a psychologist.
Autisme Info Service	https://www.autismeinfoservice.fr/	Provides active listening and information services via phone and email, tailored to autistic individuals, their families and professionals. Peers offer guidance on themes including autism (prevalence, signs, definition), screening, disability support, administrative processes, school inclusion and employment.
Les Bobos à la ferme	https://www.parents‐aidants.fr/	Open discussion about parenting, create connections and find meaning.
Café autisme	https://www.cafeautisme.com/team‐3	Weekly parent support group.
Centre ressources autisme Normandie‐Seine Eure (CRANSE)	http://cra‐normandie‐seine‐eure.fr/	Mission is to provide reliable advice, support and information to families; organize and facilitate cafés parents (monthly; various centres; minimum 5 families, no cost) in the territory of the Seine‐Maritime and Eure; accompany families in their training; listen to and report on the needs of families; provide referral to professionals as needed. Values include the expertise of parents, sharing their experience and knowledge, reduce isolation, create connections between caregivers and respond to daily needs. Training for caregivers on topics related to daily care and administrative tasks (e.g., MDPH application). One professional (paid) caregiver peer support person is on staff.
Cœur de Maman	https://coeurdemaman.net/	In‐person 2‐h meetings 7 or 8 times per year. Focus on sharing experience as a mother rather than child's challenges.
Constellation	https://www.constellation44.fr/page/817895‐accueil‐et‐soutien‐individuel	Individual meeting with a peer parent at home, organization office or other location. Peers offer listening, shared experiences and information regarding rights, medico‐social services, recreation and more. Support to identify solutions, take administrative steps, identify priorities and accompany the parent to important meetings or appointments. The relationship remains available as needed. Other supports from the association include respite and promoting parent expertise through training of professionals.
Espairs	https://www.espairs.org/	Individual meeting with peer at the organization's office, home, partner location or over the phone. Meet to discuss lived experience and knowledge, address barriers to care and rehabilitation, support destigmatization, strengthen the power to act, support therapeutic alliance, support connection to other supports, support development of crisis plans. For individuals with psychiatric or neurodevelopmental diagnoses or their caregivers. Organization also supports and advocates for professional training as a peer support person.
L'Esperluette	https://lesperluette31.com/cafe‐des‐parents/	Occasional parent and sibling (6‐ to18‐year‐olds; 8 participants per group) support groups offered at the same time. Sibling group: discuss questions, understanding and valuing role of the sibling, expressing needs, finding and sharing resources, ideas and tips. Parent group: offered open ended and thematic discussions. Resources are offered and sharing of parental expertise is encouraged.
Halte pouce	https://www.halte‐pouce.fr/pole‐familles	Bimonthly parent support group meetings limited to 15 participants. Open ended conversations to exchange points of view, ideas and life circumstances. Volunteers present to provide childcare.
Handimômes cambrésis	https://www.handimomes.fr/2024/02/28/planning‐de‐fevrier‐a‐juin/	Occasional group meeting. No details provided.
Ikigai	https://www.association‐ikigai.org/	Monthly parent meeting with a focus on educational and social inclusion.
Soliane	https://www.associationsoliane.fr/services‐pour‐les‐parents/	Once per trimester parent support group. Professionals sometimes invited to offer workshops or presentations and answer questions. Open discussion surrounded theme suggested by the association. Childcare provided.
Tom enfant phare	https://www.tomenfantphare.fr/les‐activites/	Weekly meeting for parents, professionals and others to meet to discuss issues, experiences and resources related to disability. During some meetings, specific themes are discussed or workshops are offered with ‘accompagnants sensibilisé au handicap’ (*disability‐aware support workers*).

The publicly available descriptions of four caregiver peer support programmes in France stood out as particularly well elaborated. First Constellation, inspired by the Quebec‐based not‐for‐profit organization ‘Étoile de Pacho’, articulated their values related to peer support processes. They emphasize the creation and maintenance of the peer relationship. Furthermore, they note that their peer supporters receive training and payment in their role.

Another organization, CRANSE, has created a paid peer support role, offering regular group meetings and the possibility of individual support. Their reported values emphasize parental expertise, sharing experiences, reducing isolation, creating connections among caregivers and addressing daily needs. Constellation and CRANSE are two of the few programmes that offer one‐on‐one peer support in addition to group meetings and other activities.

Similarly, Espairs describes their values and details offers of individual peer support to empower caregivers, share experiences, address barriers to care and rehabilitation, reduce stigma, strengthen therapeutic alliance, connect to supports and develop crisis plans. The organization also supports and advocates for professional training in the peer support role.

Finally, Autisme Info Service (2024), the only organization included in this study to report a national mandate with specific expertise on autism or NDD, offers general information as well as active listening phone and email services for autistic persons, their entourage and professionals. The organization does not publicly advertise as a caregiver peer support service; however, a representative indicated that their paid employees are all caregivers (Autisme Info Service, personal communication, 24 June 2024). Employees receive training and supervision related to active listening and education on various topics related to NDDs. A partnership with a specialized lawyer allows for addressing employees' questions about users' rights. Additionally, peer support among employees is organized to handle complex situations with service users.

## 3 Discussion

Our study reports on 16 organizations offering peer‐based support to caregivers of autistic children in France, contributing important insights into the nature and scope of these services. This study aimed to address three research questions. First, we sought to understand what caregiver peer supports are available for families of autistic children in France and their expressed purposes. Many of these services aim to provide informal emotional and informational support, often in group‐based settings, to reduce caregiver isolation and share practical advice (Tables 1 and 2). The emphasis of these programmes varied widely, with some focused on advocacy for social inclusion in education (e.g., Ikigai), others on sharing caregiver experiences (e.g., Cœur de Maman), and some providing little information about the objectives of their support groups.

Second, we examined the target audiences of these supports. While several programmes specifically served caregivers of children with autism or broader neurodevelopmental disabilities, the majority were open to families of children with any disability. These supports spanned 14 of France's 101 departments (geographic administrative divisions). Beyond these regional initiatives, two programmes—Cœur de Maman and Autisme Info Service—offered support at a national level, extending their reach to caregivers across the country.

Finally, we examined the structural and operational characteristics of caregiver peer support programmes. Group‐based and in‐person supports dominate the landscape, with few programmes offering individual peer support options. Key structural features, such as whether peer supporters are paid or volunteer, were inconsistently reported, with only eight organizations providing this information. Similarly, few programmes described mechanisms for onboarding, training or ongoing supervision—components critical to the sustainability and effectiveness of peer support roles.

Caregiver peer support for families of autistic children and other NDDs remains an underdeveloped aspect of care for this population in France. Our study highlights the limited nature of descriptions regarding how peer support is delivered, who delivers it, under what conditions it is provided and how peers are supported in their roles. Clear and detailed articulation of these components is essential for several reasons: It enables potential users and professionals in medico‐social and educational sectors to better understand and collaborate with peer support services; it fosters growth and sustainability by encouraging the development of comprehensive and replicable programme models; and it helps engage potential caregiver peer supporters, particularly those who may lack direct connections to existing organizations that offer peer support. For instance, McCrossin and colleagues describe a caregiver peer support programme that successfully built sustainability through community relationship‐building and integration within service networks, providing a model for how these elements can be effectively addressed (McCrossin et al. 2022; McCrossin and Lach 2023).

Training programmes like iMind's university certificate (iMind 2022) in Lyon provide a promising example of how to equip caregiver peer supporters with the skills needed to address the emotional, administrative, legal and systemic challenges faced by families. On a part‐time basis over 8 months, trainees are provided with regular opportunities to practice their skills through simulation exercises and engaging in clinical discussions. While this training programme is comprehensive, its significant time and financial commitments, as well as the requirement of a bachelor‐level degree, could present barriers to accessibility for some individuals. Similarly, the association Unafam also offers caregiver peer support training, targeting those supporting persons with psychiatric disorders rather than autism or related neurodevelopmental disorders (Unafam 2024).

In addition to these professional training programmes, other efforts are emerging to strengthen the caregiver peer support infrastructure in France. The Groupement national centres resources autism (GNCRA), which is a national body supporting local autism resource centres, has developed an online platform for training caregivers. Although this training primarily focuses on accessing and delivering autism care rather than on the role of peer support relationships (‘Formation des Proches Aidants (FPA)’ 2024), it highlights an important step towards formalizing caregiver roles within care systems. Furthermore, the professional caregiver peer support role within CRANSE, an autism resource centre included in our results, exemplifies a replicable model that could be expanded across other autism resource centres in France. These initiatives, while promising, underscore the need for continued investment in caregiver training and support to ensure sustainable and accessible peer support services.

Only four organizations in our study reported having a paid peer support role, reflecting an ongoing debate over the professionalization of peer support. Both paid and volunteer models have justifiable arguments for and against them. In the mental health literature, concerns have been raised about the impact of payment on the reciprocal nature of peer relationships (Gillard 2022). Paid roles may also lead to role ambiguity within care teams and unclear boundaries between providing and receiving support. Nevertheless, providing peer support is a form of labour. It is often emotionally taxing (Shilling et al. 2015a) and requires individuals to reflect on their challenging and potentially traumatic experiences as caregivers (McCrossin et al. 2022). Expecting caregivers, who already experience heightened stress (Hayes and Watson 2013), to take on additional unpaid labour may place an undue burden on this population. Compensating caregivers for the unique value they contribute to the care landscape recognizes their expertise, but implementing paid roles requires careful consideration. Ensuring the sustainability and effectiveness of paid caregiver peer support roles while maintaining the authenticity of peer relationships remains a key challenge for the field.

## 4 Limitations and Future Research

This study provides a valuable starting point for understanding caregiver peer support for families of autistic children in France, though a key limitation should be noted. The study relied exclusively on publicly available data, which may not fully reflect how peer support operates in practice or how it is experienced by caregivers. Consequently, some organizations offering caregiver peer support may have been missed, particularly those with limited online presence. With only one primary researcher conducting the search, the study was unable to pursue more comprehensive outreach, such as directly contacting all parent associations. Such outreach might have uncovered informal or unstructured peer support activities not documented online, along with critical programme details, such as frequency and duration of offerings, whether groups are open or closed, programme longevity or existing evaluations of programme impact. Nonetheless, accessible and clear publicly available information is crucial for caregivers, who often rely on easily discoverable resources due to the demands of caregiving (d'Eyssautier 2023). Focusing on publicly available data reflects this practical reality: To effectively reach families, organizations must ensure their services are visible and accessible.

Despite its limitations, this study has several strengths. The environmental scan used an iterative search process informed by expert feedback to identify and map caregiver peer support programmes across France. The findings provide an initial overview of the current landscape, highlighting both the diversity of available supports and key gaps in relation to their accessibility, clarity and sustainability. As one of the first efforts to document caregiver peer support in the French context, this work offers a useful starting point for future research and programme development.

Future research should build on this foundation by addressing the identified gaps and expanding our understanding of caregiver peer support. Specifically, research is needed to explore the actual benefits of caregiver peer support for families, including its impact on caregiver well‐being, family functioning and child outcomes. Studies should also investigate whether certain programme characteristics, such as format (e.g., group vs. individual), payment structures (paid vs. volunteer) and training protocols, influence the effectiveness of peer support. Additionally, examining the themes most frequently addressed in peer support settings—such as advocacy, emotional support or navigating services—could help identify areas of unmet need and inform the development of tailored interventions.

Another important avenue for future research involves collecting stakeholder perspectives, including those of caregivers, peer supporters and professionals, to better understand how caregiver peer support operates in practice. Qualitative studies could shed light on the unique aspects of family peer support in France, its impact on families, its integration into the care landscape and its influence on other professional care providers. By addressing these questions, future research can help inform strategies to expand and sustain caregiver peer support as a valuable component of autism care.

Finally, addressing the gaps in clarity of support description and terminology standardization identified in this study will be important for advancing caregiver peer support in France. Efforts should be made to promote the term ‘pair‐aidance familiale’ (family peer support) (iMind 2022), to distinguish this work from other types of peer support, such as that among autistic individuals, and to encourage its recognition as a distinct and valuable component of care for the whole family. Standardizing terminology could improve accessibility for caregivers and professionals alike while fostering growth and sustainability in the field.

## 5 Conclusions

Caregiver peer support is gaining momentum internationally as a valuable resource for families of children with NDDs, including autism. However, in France, this role remains underutilized despite its potential to address significant gaps in the care landscape. The findings of this study highlight the diversity of caregiver peer support programmes, from informal group‐based gatherings such as ‘café des parents’ to structured models like those offered by Constellation and CRANSE. These programmes vary widely in their purpose and implementation, with some focusing on advocacy or emotional support and others emphasizing individualized assistance or system navigation. Notably, only a few programmes provided detailed information on training, payment or sustainability mechanisms, underscoring the need for greater clarity and standardization. Promising initiatives, such as iMind's caregiver peer support training programme in Lyon, offer an example of how comprehensive training can equip peer supporters with the skills needed to address these families' challenges effectively. Leveraging expertise from programmes like iMind's could help establish more consistent and sustainable practices across caregiver peer support services in France.

The programmes examined in this study demonstrate the potential of caregiver peer support to address unmet needs, foster resilience and enhance family well‐being. However, the variability in descriptions and practices suggests that further investment is needed to expand and strengthen these services. Promoting caregiver peer support as a distinct and valuable component of autism care in France—through the adoption of standardized terminology, increased visibility and professionalized training—could foster growth and improve accessibility.

Future research should build on these findings by exploring the impact of caregiver peer support programmes on family outcomes and evaluating the effectiveness of various implementation strategies. With a growing recognition of the value of caregiver expertise, caregiver peer support has the potential to play a pivotal role in advancing family‐centred care in France.

## Author Contributions

J.M. led the project, including conceptualization, methodology design, data analysis and manuscript drafting. He also secured funding for the study. L.L. contributed to the study's conceptual framework and critically reviewed and edited the manuscript. S.B. participated in the validation of study findings and content review and provided feedback on the manuscript. E.C. provided resources and supervision for project implementation, contributed to conceptualization and reviewed and edited the manuscript.

## Ethics Statement

As this study was based on publicly available information, no ethics approval was sought.

## Conflicts of Interest

The authors declare no conflicts of interest.

## Data Availability

The data supporting the findings of this study were obtained from publicly available websites and resources, as well as through consultations with local academic and community experts. Data extracted from websites were publicly accessible at the time of collection and are cited within the manuscript. Additional details from private communications with organizations have been anonymized to protect the privacy of individuals involved. Requests for further information on specific data or additional inquiries can be directed to the corresponding author.

## Appendix I Internet Search Details

**Table:**

Category	English search terms	French search terms
Primary keywords	‘caregiver peer support’ OR ‘parent peer support’ OR ‘family peer support’	‘soutien par les pairs pour les aidants’ OR ‘soutien par les pairs pour les parents’ OR ‘soutien familial par les pairs’
	AND ‘autism’ OR ‘autistic children’ OR ‘autism spectrum disorder’ OR ‘neurodevelopmental disabilities’ OR ‘neurodisabilities’	AND ‘autisme’ OR ‘enfants autistes’ OR ‘troubles du neuro‐développement’ OR ‘neurodisabilités’
	AND ‘France’ OR ‘French’	AND ‘France’ OR ‘français’
Secondary keywords	‘support groups’ OR ‘mutual aid’ OR ‘parent associations’	‘groupes de soutien’ OR ‘entraide mutuelle’ OR ‘associations de parents’ OR ‘groupe de parole’ OR ‘café de parents’
	AND ‘resources’ OR ‘services’ OR ‘organizations’ OR ‘interventions’	AND ‘ressources’ OR ‘services’ OR ‘organisations’ OR ‘interventions’
	AND ‘caregiver well‐being’ OR ‘family support’	AND ‘bien‐être des aidants’ OR ‘soutien familial’

Search engine used: Google.

## Appendix II Resource Directories and Caregiver Organizations

**Table:**

Website	Description
Autisme France https://www.autisme‐france.fr/	A national autism association advocating for the rights of individuals with autism. Provides support to families and promotes access to evidence‐based interventions.
Autisme Info Service https://www.autismeinfoservice.fr/	A national helpline and online resource dedicated to autism information, support and referrals for families and individuals with autism.
CRAIF https://www.craif.org/	The Ile‐de‐France Autism Resource Center, offering a wide range of autism‐related information, resources and guidance for families, caregivers and professionals.
Enfant Différent https://www.enfant‐different.org/	A comprehensive resource hub providing information and guidance for families of children with disabilities, including neurodevelopmental conditions. Covers rights, education and support services.
Métropole Aidante https://www.metropole‐aidante.fr/	A regional initiative in the Lyon metropolitan area providing support, resources and information for caregivers of individuals with various disabilities.
Sesame Autisme https://sesame‐autisme.com/	Organization supporting autistic individuals and their families through advocacy and services.
Tamis Autisme https://www.tamis‐autisme.org/	A platform focused on autism advocacy and resource sharing. Offers tools for families and professionals to navigate autism‐related support and services.
Unapei https://www.unapei.org/	National network advocating for individuals with intellectual and cognitive disabilities and their families.

## Appendix III Data Extraction Tool

**Table:**

Resource	
Organization name	Name of organization offering organized caregiver peer support.
Website	Website or social media link.
Target condition or diagnosis	e.g., ASD, neurodevelopmental disabilities, intellectual disabilities, general disability
Target location	Region, department, country‐wide
Name of peer support service	Specific service name
Description of service	Description of the group, network or service offered by the organization.
Meeting location	e.g., online, in‐person
Structure of service	e.g., peer matching/individual support from peer, weekly/monthly group meetings, reciprocal, voluntary or paid facilitator
Support for peers	Onboard training, continuing education, consultation/supervision with professional or experienced peer mentor
